# Sleep quality in mechanically ventilated patients: comparison between NAVA and PSV modes

**DOI:** 10.1186/2110-5820-1-42

**Published:** 2011-09-28

**Authors:** Stéphane Delisle, Paul Ouellet, Patrick Bellemare, Jean-Pierre Tétrault, Pierre Arsenault

**Affiliations:** 1 Service des soins intensifs, Hôpital du Sacré-Cœur de Montréal, Montréal, Québec, Canada; 2 Département de médecine familiale et d'urgence, Université de Montréal, Montréal, Québec, Canada; 3 Département des sciences cliniques, Université de Sherbrooke, Sherbrooke, Québec, Canada; 4 Département de chirurgie, Centre hospitalier universitaire de Sherbrooke, Sherbrooke, Québec, Canada; 5 Service des soins intensifs, Hôpital régional d'Edmundston, réseau de santé Vitalité, Edmundston, Nouveau-Brunswick, Canada

## Abstract

**Background:**

Mechanical ventilation seems to occupy a major source in alteration in the quality and quantity of sleep among patients in intensive care. Quality of sleep is negatively affected with frequent patient-ventilator asynchronies and more specifically with modes of ventilation. The quality of sleep among ventilated patients seems to be related in part to the alteration between the capacities of the ventilator to meet patient demand. The objective of this study was to compare the impact of two modes of ventilation and patient-ventilator interaction on sleep architecture.

**Methods:**

Prospective, comparative crossover study in 14 conscious, nonsedated, mechanically ventilated adults, during weaning in a university hospital medical intensive care unit. Patients were successively ventilated in a random ordered cross-over sequence with neurally adjusted ventilatory assist (NAVA) and pressure support ventilation (PSV). Sleep polysomnography was performed during four 4-hour periods, two with each mode in random order.

**Results:**

The tracings of the flow, airway pressure, and electrical activity of the diaphragm were used to diagnose central apneas and ineffective efforts. The main abnormalities were a low percentage of rapid eye movement (REM) sleep, for a median (25th-75th percentiles) of 11.5% (range, 8-20%) of total sleep, and a highly fragmented sleep with 25 arousals and awakenings per hour of sleep. Proportions of REM sleep duration were different in the two ventilatory modes (4.5% (range, 3-11%) in PSV and 16.5% (range, 13-29%) during NAVA (*p = *0.001)), as well as the fragmentation index, with 40 ± 20 arousals and awakenings per hour in PSV and 16 ± 9 during NAVA (*p *= 0.001). There were large differences in ineffective efforts (24 ± 23 per hour of sleep in PSV, and 0 during NAVA) and episodes of central apnea (10.5 ± 11 in PSV vs. 0 during NAVA). Minute ventilation was similar in both modes.

**Conclusions:**

NAVA improves the quality of sleep over PSV in terms of REM sleep, fragmentation index, and ineffective efforts in a nonsedated adult population.

## Background

**S**leep is severely disturbed in mechanically ventilated ICU patients [1-3]. Sleep alterations are known to have deleterious consequences in healthy subjects, but the paucity of data in the literature [4-7] makes it difficult to determine the impact of sleep abnormalities in ICU patients. Intensive care unit (ICU) patients present disrupted sleep with reduced sleep efficiency and a decrease in slow wave sleep and rapid eye movement (REM) sleep [8-10]. Furthermore, polysomnographic studies performed on mechanically ventilated ICU patients have demonstrated an increase in sleep fragmentation, a reduction in slow-wave and REM sleep, and an abnormal distribution of sleep, because almost half of the total sleep time occurred during the daytime [11-13]. In the Freedman et al. study [14], noise was considered a nuisance for the patients questioned; the most annoying noises were alarms and caregivers' conversations. When the same authors simultaneously recorded noise and microarousal, they identified an association between arousal and noise in only 11-17% of the cases [11]. This percentage is confirmed by Gabor et al. [3] where 21% of the arousal interruptions were explained by loud noises and 7% to patients' care. Seventy-eight percent of the microarousals were not associated with environment noises, suggesting other causes, such as patient/ventilator asynchrony [3,14].

The effects of assist control ventilation (ACV) and pressure support ventilation (PSV) on sleep fragmentation have been examined in critically ill patients receiving mechanical ventilation [15], where PSV mode was associated with increases in the number of central apneas and subsequent sleep fragmentation compared with AVC. Furthermore, the study suggested that PSV by itself or an excess of ventilator assistance with PSV could have caused such sleep alterations. Indeed, ventilatory settings adjusted during wakefulness may become excessive during sleep, as the patients' ventilatory demand is reduced while asleep [16]. Whether these results can be explained by the ventilatory mode itself or how it was adjusted is an important issue, because hyperventilation and patient ventilator asynchrony may result from PSV as well as ACV in mechanically ventilated ICU patients [17]. Fanfulla et al. [18] compared two ventilatory settings in nine patients under long-term PSV for neuromuscular disease. The initial setting was set according to clinical parameters, and the second setting was adjusted with measurement of esophageal pressure (physiological setting) to optimize patient effort. The physiological setting improved the duration and quality of sleep, decreased episodes of apnea, and the amount of inefficient efforts for ventilator triggering [18]. The level of pressure support and PEEP tended to decrease, with a lowering of intrinsic PEEP and patient-ventilator asynchronies. A recent study by Cabello et al. [19] compared the impact of three modes of ventilation (AVC, PSV, and SmartCare™) on the quality of sleep in alert and nonsedated patients, and no difference for the architecture, fragmentation, and duration of sleep was found among the three modes.

Our hypothesis is that NAVA ventilation is superior to PSV by allowing optimal patient-ventilator synchrony and thereby decreasing sleep fragmentation.

## Methods

This study was approved by the Ethics Committee of the Hôpital du Sacré-Coeur de Montréal, and patients or their surrogates gave written informed consent.

### Patients

This physiologic study was conducted in a 22-bed medical ICU during a 12-month period. The weaning phase of mechanical ventilation was chosen because patient-ventilator asynchrony is common when patients are spontaneously triggering breaths. The inclusion criteria required that the patient was conscious, free from sedation and opiate analgesia for ≥ 24 hours, and ventilated in PSV mode with an FIO2 < 60%, PEEP = 5 cmH_2_O, and SpO2 ≥ 90%. Exclusion criteria consisted of the presence of a central nervous system disorder, Glasgow Coma Scale score < 11, hemodynamic instability, renal and/or hepatic insufficiency, and ongoing sepsis.

### Methods

**Figure 1:**
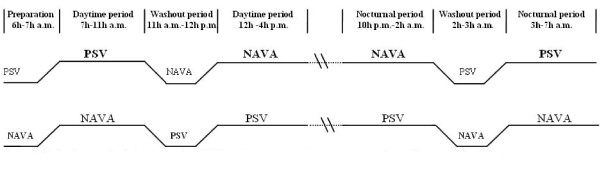
**Patients were studied for a period of 4 hours for each recording sequences and for more than 19 consecutive hours**.

All patients were ventilated through an endotracheal tube or a tracheostomy; once they met the inclusion criteria, they were connected to a Servo *i *ventilator (Maquet critical Care, Sölna, Sweden), equipped with a neurally adjusted ventilator assist system (NAVA). The electrical activity of the diaphragm (EAdi) is captured with the EAdi catheter (Maquet Critical Care, Sölna, Sweden) consisting of a 16-Fr gastric tube equipped with electrodes. End-tidal CO_2 _was monitored with the Servo-i Volumetric CO2 module. The two different ventilatory modes were delivered in a randomized order using a closed-envelope technique during four periods of 4 hours: a daytime period from 7 to 11 a.m. and 12 to 4 p.m., and a nocturnal period from 10 p.m. to 2 a.m. and 3 to 7 a.m. To prevent possible data contamination from the previous mode of ventilation, a 1-hour washout period after a ventilator change was introduced before data acquisition (Figure 1; Study Protocol).

During periods of wakefulness, PSV and NAVA were clinically adjusted by the attending physician to obtain a tidal volume of 8 mL/kg of predicted body weight and a respiratory rate ≤ 35 breaths/min. For both modes of ventilation, inspiratory triggering sensitivity was set at thresholds that would not allow auto-triggering for both modes of ventilation: 0.5 mV in NAVA and 5 in PSV.

EEG was recorded from standard locations: left frontal/right mastoïd reference (F3/M2 or F3/A2), right frontal/left mastoid reference (F4/M1or F4/A1), left central/right mastoïd reference (C3/M2 or C3/A2), right central/left mastoïd reference (C4/M1 or C4/A1), left occipital/right mastoïd reference (O1/M2 or O1/A2), and right occipital/left mastoïd reference (O2/M1or O2/A1), according to the International 10-20 System for electrode placement [20]. The standard reference used was the left mastoid lead [20]. Two electro-oculogram and three chin electromyogram leads were used to score REM and non-REM sleep. The electroencephalogram, the right and left electro-oculogram, and the submental electromyogram signals were amplified and recorded in the data acquisition system (Alice 5 polysomnography system using Alice^® ^Sleepware™ 2.5 software, Respironics, Nantes, France).

Sleep recordings were manually read and scored by an independent pulmonologist blinded to the study, using the criteria of Rechtschaffen and Kales [21,22] and the criteria of the American Sleep Disorder Association for arousals and awakenings [23,24]. Diagnosis of central apnea was based on international recommendations [24]. The diagnosis of central apnea is characterized by absent breathing and respiratory effort for a period of at least 10 seconds. Arousals and awakenings were considered secondary to apnea when occurring within three cycles and/or 15 sec after a respiratory event [25,26]. Ineffective efforts were defined as an inspiratory effort observed by a peak electrical activity of the diaphragm (EAdi peak) without a simultaneously triggered ventilator cycle. Airflow, Paw, and EAdi were acquired from the ventilator through a RS232 interface at a sampling rate of 100 Hz, recorded by a dedicated software (Nava Tracker V. 2.0, Maquet Critical Care, Sölna, Sweden), and an analyzer using software Analysis V 1.0 (Maquet Critical Care) and a customized software based for Microsoft Excel. An arousal or awakening event was considered secondary to ineffective triggering when it occurred within 15 seconds after the asynchrony [19].

Noise was measured with a portable noise meter at the level of patient's head (Quest Technologies, Oconomowoc, WI). Arousals and awakenings were associated with the noise when they occurred 3 seconds after or within noise increase ≥10 dB [3,11]. Inspiratory trigger delay was calculated as the time difference between the onset of EAdi peak and Paw inspiratory swings. Cycling-off delay was calculated as the time difference between the end of the inspiratory EAdi peak deflection and the onset of expiratory flow.

### Statistics analysis

Statistical analysis was performed using SPSS statistical software (SPSS 17.0). Continuous variables were expressed as median (25^th^-75^th ^percentile) or mean ± SD. Data were compared using the general linear model for repeated measures (GLM). The small sample of patients led us to use Wilcoxon's *t *test for paired samples, and the *p *values for multiple comparisons were corrected for the Bonferroni inequality. A two-tailed *p *value < 0.05, corrected as needed, was retained to indicate statistical significance.

## Results

### Patients

Fourteen patients were selected and none were excluded during the study. Their main characteristics are shown in Table 1. Acute respiratory failure was the most frequent reason to initiate mechanical ventilation in ten patients, postoperative complications in three patients, and septic shock in one patient.

**Table 1:** Characteristics of patients

Characteristics of patients	
Sex (M/F)	(8/6)
Age (yr ± SD)	64 ± 11
SAPS II ± SD	46 ± 12
Duration of MV (days ± SD)	17 ± 9
Tracheotomy (%)	2 (14)
Cause for initial MV (%)	
Acute respiratory failure	10 (71.5%)
Postoperative complication	3 (21.5%)
Septic shock	1 (7%)

M = male; F = female; SAPS = Simplified Acute Physiology score; MV = mechanical ventilation.

### Sleep recordings

All patients completed the study, and recordings were well tolerated. Individual sleep data are shown in Table 2. The median total sleep time was 564 (range, 391-722) minutes. The median sleep efficiency (i.e., the percentage of sleep during the study) was 59% (range, 41-75%). The main abnormalities observed on each patient were a diminished percentage of REM sleep, counting for only 11.5% (range, 8-20%) of total sleep time, and a high fragmentation index with 25 arousals and awakenings per hour (range, 18-51). Although interindividual variability was large, the median quantity of slow-wave sleep (stages 3 and 4 or NREM3 stage) was normal, with a median of 18.5 (range, 11.5-22; Table 2).

**Table 2:** Sleep architecture and fragmentation during the study (16 hours)

Patient	Stage 1 (%)	Stage 2 (%)	Stages 3 and 4 (%)	Rapid eye movement (%)	Fragmentation index
1	5	72.5	19.5	2.5	23.5
2	2.5	67	22.5	7	35.5
3	4	61	24.5	9	30.5
4	10	57	11	20	68
5	9	61	24	5.5	16.5
6	6	57	24	12.5	15
7	5	63	22	7.5	13.5
8	11	66.5	9	10.5	64.5
9	5.5	58	17.5	19	15.5
10	11	60.5	9.5	17	56.5
11	3	66	21	9	26
12	3.5	61.5	13	22	23
13	5.5	60	13	21.5	22
14	10	59	10.5	20.5	67.5
Median [25-75^th ^percentiles]	5.5 [4-10]	61 [59-65]	18.5 [11.5-22]	11.5 [8-20]	25 [18-51]

### Ventilatory modes and sleep distribution

**Figure 2:**
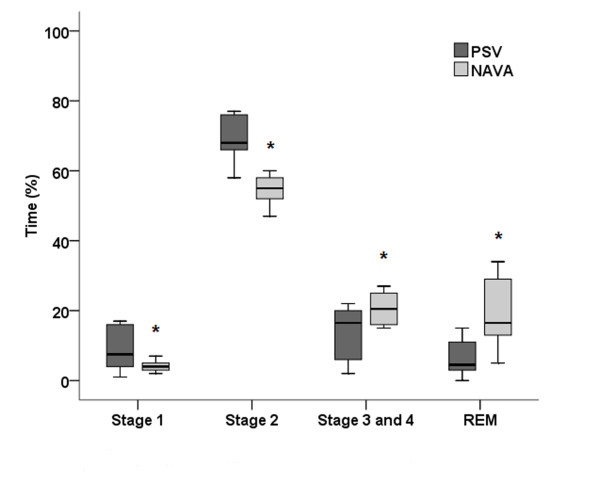
**Sleep stages (percent of total sleep) during the two ventilator modes: pressure support ventilation (PSV), and neurally adjusted ventilatory assist (NAVA)**. REM = rapid eye movement.

Sleep efficiency and architecture appeared very different for both modes of ventilation (NAVA and PSV). Stage 1 (NREM1 stage) lasted longer during PSV compared with NAVA 7.5% (range, 4-15%) vs. 4% (range, 3-5%; *p *= 0.006). Stage 2 (NREM2 stage) also lasted longer in PSV than NAVA 68% (range, 66-75%) vs. 55% (range, 52-58%; *p *= 0.001). Stage 3-4 (NREM3 stage) was shorter in PSV as opposed to NAVA 16.5% (range, 17-20%) vs. 20.5% (range, 16-25%; *p *= 0.001). REM stage (R stage) was much shorter in PSV than in NAVA 4.5% (range, 3-11%) vs. 16.5% (range, 13-29%; *p *= 0.001). The fragmentation index was different between the two ventilation modes, with 40 ± 20 arousals and awakenings per hour in PSV and 16 ± 9 during NAVA (*p *= 0.001; Figure 2 Sleep stage (percent of total sleep) during two ventilatory modes; Table 3).

**Table 3:** Comparison of sleep quality between the ventilatory modes

	PSV	NAVA	*p*
Stage 1, %	7.5 [4-15]	4 [3-5]	0.006*
Stage 2, %	68 [66-75]	55 [52-58]	0.001*
Stage 3 and 4, %	16.5 [17-20]	20.5 [16-25]	0.001*
REM, %	4.5 [3-11]	16.5 [13-29]	0.001*
Fragmentation index, (n/h)	33.5 [25-54]	17.5 [8-21.5]	0.001*
Sleep efficacy, %	44 [29-73.5]	73.5 [52.5-77]	0.001*

PSV = pressure support ventilation; NAVA = neurally adjusted ventilatory assist; REM = rapid eye movement; Fragmentation Index = number of arousals and awakenings per hour of sleep; Sleep efficiency = duration of sleep/total duration of recording.

Values are expressed as median [interquartile range].

*p < 0.05.

Minute ventilation did not significantly differ between PSV and NAVA with median values of 9.8 L/min (range, 8.0-10.9), and 9.6 L/min (range, 7.5-11.0) respectively (*p *= 0.51). The median respiratory rates were 17 breaths/min (range, 14-21), and 20 breaths/min (range, 15-23) during PSV and NAVA (*p *= 0.14). Median tidal volume was 420 mL (8.1 mL/Kg of predicted body weight; range, 375-479 mL), and 378 mL (7.3 mL/Kg of predicted body weight; range, 370-448 mL) during PSV and NAVA, respectively (*p *= 0.36). The mean PSV level was 15 ± 5 cmH_2_O, and the mean NAVA level was 1.6 ± 1.4 cmH_2_O/μV. Positive end-expiratory pressure was kept at 5 cmH_2_O for all patients.

### Apneas and ineffective efforts

Ten of the 14 patients presented sleep apnea, and 11 exhibited ineffective efforts. The mean index of sleep apneas (number of apneas per hour of sleep) was 10.5 ± 11 apneas during PSV and 0 during NAVA (*p *= 0.005) and ineffective efforts (number of ineffective efforts per hour of sleep) was 24 ± 23 ineffective efforts during PSV and 0 during NAVA (*p *= 0.001). Over-assistance during sleep is sensed on the previous three cycles preceding central apnea. Tidal volume and minute ventilation increased, whereas ETCO2 and EAdi decreased over the three cycles preceding central apnea Table 4.

**Table 4:** Oscillatory behaviour of various ventilator parameters for stages 3-4 with PSV mode of ventilation

Respiratory variables	Baseline	Pre-apneas PSV
V_T _(mL)	425 ± 67	585 ± 70
RR (breath/min)	13 ± 2	12 ± 1
VE (L/min)	5.2 ± 0.5	6.8 ± 0.8
ETCO_2 _(mmHg)	46 ± 1.4	42 ± 1.0
EAdi (mVolt)	15 ± 4	10 ± 2

V_T _= tidal volume; RR = respiratory rate; VE = minute ventilation; ETCO_2 _= end-tidal carbon dioxyde; PSV = pressure support ventilation.

### Trigger delay and cycling-off delay

During N-REM sleep in PSV, the trigger delay increased on average by 80 ± 26 (msec) during stage 1 versus 158 ± 42 (msec) during stage 3 and 4. The expiratory trigger (cycling-off) increased in PSV by 158 ± 103 (msec) and 258 ± 87 (msec) during stage 1 and stages 3 and 4, respectively. In NAVA, the trigger delay remained stable during sleep, 68 ± 24 (msec) during stage 1 and 72 ± 32 (msec) during stages 3 and 4. The expiratory trigger also remained stable in NAVA: 39 ± 28 (msec) during stage 1 and 41 ± 34 (msec) during stages 3 and 4.

### Noise

In ICU, we recorded the average baseline ambient noise level and evaluated arousals from this baseline to a peak noise level ≥ 10 dB above ambient noise level. The mean noise level was recorded at 64 ± 8 dB, with the peak level recorded at 111 dB and the minimal level at 52 dB. No differences were observed between the two different ventilatory modes concerning the index of fragmentation associated with noise: 7.5 ± 3 during PSV and 6 ± 3.5 during NAVA (*p *= 0.19). These data indicate that 18% during PSV and 21% during NAVA of the fragmentation was associated with sudden increases in noise.

### Sleep distribution among study periods

**Figure 3:**
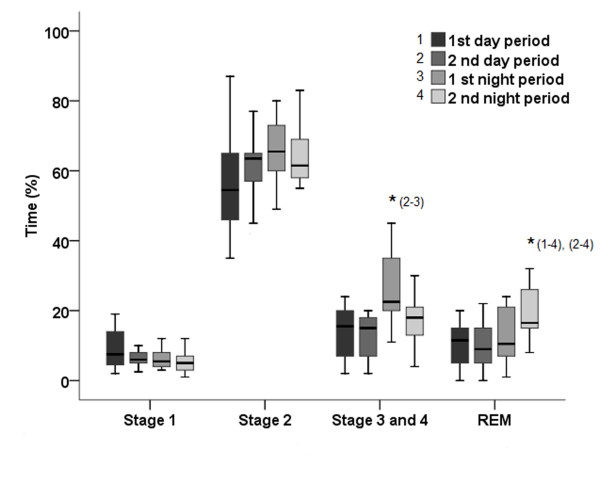
**First daytime period (7 h-11 h a.m.), second daytime period (12 h-4 h p.m.), first nighttime period (10 h p.m. to 2 h a.m.) and second nighttime period (3 h-7 h a.m.)**.

The cross-over pattern was balanced with an equal number of patients from each sequence initiating the rotation. Independent of the ventilatory mode, sleep efficiency and sleep architecture had a significantly different distribution based on the study period considered (Figure 3--sleep stage (percent of total sleep) during the four daily time periods). Sleep efficiency was the same in the two daytime periods (2 periods during the day): 52% (range, 26-67%) during the first day period (7 h-11 h a.m.) and 51.5% (range, 27-67%) during the second day period (12 h-4 h p.m.; *p = *0.18). Sleep efficiency also did not differ between the two night periods: 65.5% (range, 37-82%) during the first night period (10 h p.m.-2 h a.m.) and 65% (range, 45-82.5%) during the second nighttime period (3 h-7 h a.m.; *p *= 0.11).

There was no statistical difference between stage 1 and 2 recording periods. A greater duration of slow-wave sleep (stage 3-4) was found during the first nocturnal period with a median percentage 22.5% (range, 20-33.5%) vs. 15.5% (range, 7-19.5%) during first day period (*p *= 0.03), vs. 15% (range, 7-18%) during second day period (*p *= 0.01) and vs. 18% (range, 13-21%) during second nighttime period (*p *= 0.001).

The proportion of REM sleep was longer during the second nocturnal period, with a median percentage of 16.5% (range, 15-25%) vs. 11.5% (range, 5-15%) during first day period (*p *= 0.001) vs. 9% (range, 5-15%) during second day period (*p *= 0.001) and vs. 10.5% (range, 7-21%) during first nighttime period (*p *= 0.02). The fragmentation index did not differ with 26 (range, 20-65) arousals and awakenings/hour during first daytime vs. 24 (range, 19-55), 23 (range, 18-57), and 19 (range, 15-53) during the second day period and first and second night period, respectively (*p *= 0.08). Ineffective effort indexes per hour also were similar across the four periods.

## Discussion

In a study where spontaneously breathing patients were conscious and under mechanical ventilation, proportions of sleep fragmentation sleep architecture and sleep quality were positively influenced by NAVA. In the PSV mode, a low percentage of REM sleep and a high degree of fragmentation were present. NAVA showed a normal percentage of REM sleep with an important decrease in fragmentation.

Less than 15% of the sleep fragmentations in the PSV mode were attributed to apneas and ineffective efforts, whereas in NAVA, no asynchrony (no apnea and no ineffective patient efforts) were recorded. Environmental noise is responsible for 18% of the arousals and awakenings in PSV compared with 21% in NAVA, respectively.

We observed results similar to the Cabello et al. [19] study concerning the rate of fragmentation, the number of central apneas, and the number of ineffective patient efforts during PSV. Another similar finding concerned the increased percentage of REM sleep during the second nighttime period recordings. However, one major difference between our study and the Cabello study is that they did not allocate an even distribution for each of the study periods and ventilatory strategies. Also, they did not allow washout periods between the ventilatory modes, which could possibly contaminate the recordings at the beginning of the next study period. Detecting asynchronies also was different; they used the airway pressure-flow signal and the thoracoabdominal plethysmography, whereas we observed the EAdi signal.

Parthasarathy and Tobin [15] found a lower rate of sleep fragmentation during ACV compared with PSV. This was explained by the central apneas induced by over-assistance during PSV. In fact, tidal volume was much greater during PSV compared with ACV. This was validated by the addition of a dead space to the 11 patients showing central apneas, which significantly decreased the number of apneas.

In the Toublanc et al. study [27], no difference was found in terms of quantity, quality of sleep, and in terms of arousal index between the AC and a low level of PSV assistance for the whole night. Toublanc et al. found that ACV was superior in terms of percentage of slow-wave sleep but not during REM sleep [27]. It is very difficult to compare the results for PSV because of a lack of information on expiratory triggering with Evita 4, number of asynchronies (tidal volume, respiratory rate, and minute ventilation). In the Toublanc study, the majority of patients were affected with COPD and pressure support was adjusted to 6 cmH_2_O. According to Brochard et al., it is suggested that for COPD patients, the pressure support needed to overcome resistance imposed by the endotracheal tube is higher than non-COPD patients: 12 ± 1.9 vs 5.7 ± 1.5 cmH_2_O respectively [28]. In the Leleu et al. study, pressure support must be superior to 6 cmH_2_O, particularly in COPD if the intention is to compensate work of breathing imposed by the endotracheal tube, ventilator circuit, and patient effort to trigger the demand valve during pressure support [29]. A low-pressure support only allows for partial relieve of imposed work of breathing without modifying the work necessary to trigger the demand valve. In the Toublanc study, pressure support set too low in COPD patients resulted in an increase in imposed work of breathing, which can be accounted for in the decrease in SWS and REM.

The Toublanc study offers no information on expiratory triggering, which is somewhat important in COPD patients. Tassaux et al. recently have evaluated the positive impact of shortening inspiratory time in PSV on patient-ventilator asynchronies and the work of breathing in COPD patients. This study also demonstrated that the increase in expiratory trigger up to 70% of peak flow improved synchrony and decreased ineffective efforts without modifying work of breathing or minute ventilation [30].

Bosma et al. evaluated the impact on sleep with other modes of ventilation, such as the proportional assist ventilation (PAV). The objective of PAV, such as NAVA, is to improve patient ventilator synchrony by delivering ventilator assist proportional to patient effort. The study by Bosma et al. shows an improvement in the quality of sleep using PAV compared with PSV during one night sleep [31]. There are similarities between the Bosma study and ours. More specifically, PAV appeared superior to PSV in terms of decrease in arousals, improvement in sleep quality, decrease in amounts of arousals, awakenings per hour, and improved SWS and REM. With NAVA, we observed a decrease in tidal volume by up to 15% during REM sleep, which increased end-tidal CO_2 _by approximately 4 mmHg. Bosma et al. observed a tidal volume slightly more elevated in PSV compared with PAV (despite similar offloading of the work of breathing), resulting in a higher morning PaCO_2 _with PAV attributed to lower tidal volume and minute ventilation [31], thus offering perhaps a protection against central apneas. Finally, fewer patient-ventilator asynchronies were observed with PAV with fewer awakenings per hour [31].

Contrary to NAVA, PAV cannot eliminate wasted or ineffective efforts. There was a nonstatistically significant difference in ineffective triggering during inspiration; 19.6 n/hr for PSV vs. 11.6 n/hr for PAV [31]. According to Thille et al. ineffective efforts and double triggering are among the most frequent asynchronies: 85 and 13% respectively [32], which is somewhat contradictory to Bosma et al. who identify auto triggering as the most frequent asynchrony in PSV.

We observed that the absence of central apnea and ineffective efforts in NAVA do not totally explain the great improvement in the SWS and REM sleep. This improvement may be explained in part by a microanalysis of the sleep architecture. The microanalysis suggests an over-assistance with PSV during the N-REM stages, because 100% of the fragmentations in PSV occurred during this stage. The tidal volume decrease in NAVA follows the respiratory physiologic changes during sleep, whereas in PSV we find a tidal volume oscillatory behavior due to constant inspiratory efforts, independent of the sleep stage and produces sequential over-assistance during N-REM sleep leading to a decrease in end-tidal CO_2_. It is our assumption that improvement of the slow-wave sleep and REM is most probably explained by better patient comfort through better neuromechanical coupling.

During sleep, the respiratory accessory muscles (intercostals, scalene, and abdominals) decrease their muscle tone and the mechanical response of the diaphragm is, in part, spent in the production of a mechanical distortion of the chest wall, secondary to a lack of synchronization between diaphragmatic contraction and the accessory muscles. NAVA improves this mechanical distortion, whereas PSV worsens this distortion by a tidal volume oscillation (overshoot) during sleep, with a constant patient effort. Patient comfort is not only directly related to inefficient efforts and central apneas; the microanalysis showed that during N-REM sleep in PSV, the trigger delay increased during stage 1 versus during stage 3 and 4. The expiratory trigger increased in PSV during stage 1 and stages 3 and 4, respectively. In NAVA, the trigger delay remained stable during stage 1 and during stages 3 and 4. The expiratory trigger also remained stable in NAVA, during stage 1 and during stages 3 and 4. NAVA allows optimizing the neuromechanical coupling and therefore patient-ventilator synchrony [33] and allows for optimized adequacy between ventilatory load and patient breathing ability, thereby providing beneficial effects on sleep in ICU patients. It appeared to us that the EAdi tracing is much more efficient than flow and pressure tracings to detect asynchronies.

Our study has some limitations; one is the open space between patients. This study included only 14 patients, which could favor the possibility of a type II error. Patients' heterogeneity implies that patients required bedside care, such as suctioning or other care, which could perhaps influence sleep fragmentation. The study by Cabello found that suctioning was associated with < 1% arousals and awakenings [19]. The choice for a 15-second interval between asynchrony and the occurrence of arousal was chosen based on one previous study on the same topic [19]. Literature on this specific time interval to choose is very scarce. In one study, it was shown that the breathing response to a complete airway occlusion was 20.4 ± 2.3 sec during NREM and 6.2 ± 1.2 sec during REM [34]. The choice of a 15-second interval seems very reasonable but may need further investigation.

In a sleep laboratory, it is a lot easier to control the baseline ambient noise level. In a clinical environment, such as an ICU, we recorded the average baseline ambient noise level and evaluated arousals from this baseline to a peak noise level ≥10 dB above ambient noise level. There is therefore a potential for statistical inaccuracies.

The fact that we stopped sedation 24 hours before beginning the study does not imply an absence of cumulative sedation. However, every patient had a Ramsay Score of 2 or less and a Glasgow Score of 11 (the maximum score for an intubated patient).

## Conclusions

The ventilatory mode NAVA improves the quality of sleep by increasing the slow-wave sleep and REM and by decreasing fragmentation. NAVA improves patient comfort through better neuromechanical coupling during N-REM sleep, by a shorter trigger delay, and more efficient expiratory triggering. To minimize sleep fragmentation, optimal setting of pressure support level and expiratory trigger are paramount in PSV. However, proportional assistance modes of ventilation according to patient inspiratory effort, such as NAVA, appear to be a better choice to minimize sleep fragmentation.

## Competing interests

The authors declare that they have no competing interests.

## Authors' contributions

SD and PO drafted the manuscript, and PB, JPT, and PA revised the manuscript.
